# Dysregulated Plasticity in Serotonin, Galanin, and Opioid Systems Contributes to Limbic Seizure Recruitment in Wistar Audiogenic Rat

**DOI:** 10.1111/jnc.70397

**Published:** 2026-03-08

**Authors:** Tays Araújo Camilo, Evandro Valentim‐Lima, José Antônio Cortes de Oliveira, Norberto Garcia‐Cairasco, Luís Carlos Reis, João Victor Nani, André de Souza Mecawi

**Affiliations:** ^1^ Department of Biophysics, Escola Paulista de Medicina Universidade Federal de São Paulo São Paulo Brazil; ^2^ Departament of Physiology Faculdade de Medicina de Ribeirão Preto, Universidade de São Paulo Ribeirão Preto Brazil; ^3^ Department of Physiological Sciences Instituto de Ciências Biológicas e da Saúde, Universidade Federal Rural Do Rio de Janeiro Seropédica Brazil

**Keywords:** Audiogenic kindling, epilepsy, hypothalamus, limbic recruitment, neurotransmission, plasticity, Wistar Audiogenic rat

## Abstract

The Wistar Audiogenic Rat (WAR) strain is a genetically selected model of reflex epilepsy, susceptible to mesencephalic and, following chronic stimulation, limbic seizures. In this study, we examined the molecular underpinnings of this seizure progression by assessing gene expression profiles of pre‐synaptic serotonergic components in Dorsal Raphe Nucleus (DRN) and post‐synaptic receptors in the Basolateral Amygdala (BLA), Central Amygdala (CeA), and Hippocampus (HIP). Concurrently, we evaluated mRNA expression of Galanin (Gal) and Prodynorphin (Pdyn) in the Supraoptic Nucleus (SON) and their respective receptors in the BLA, CeA, and HIP. WARs and control Wistar rats underwent a ten‐day audiogenic kindling (AK) protocol, involving twice‐daily exposure to a high‐intensity acoustic stimulus to induce seizures. WARs were sub‐grouped based on their behavioral phenotype (seizure scales) into limbic‐recruited seizures (LiR) and non‐limbic‐recruited (n‐LiR). Quantitative PCR analysis of brain micropunches revealed a significant failure of adaptive plasticity in WARs. Unlike control rats, which showed a robust upregulation of serotonergic (5‐HT‐ergic) components in the DRN in response to the chronic stress of the kindling protocol, WARs had a significantly blunted pre‐synaptic response. Rats that did not show limbic seizures showed compensatory upregulation of amygdala 5‐HT receptors, a mechanism that failed in rats that developed chronic seizures. Furthermore, WARs showed elevated hypothalamic galanin but reduced limbic receptor expression. The opioid system was also imbalanced, with an increase in the pro‐convulsant mu‐opioid receptor. Critically, Pdyn expression was strongly and negatively correlated with limbic seizure severity. Collectively, these findings suggest that the progression to limbic epilepsy, already demonstrated in behavioral and EEG protocols in this model, is driven by a widespread failure of plasticity across interconnected neuromodulatory networks, rather than a single molecular defect, highlighting novel targets for therapeutic intervention.

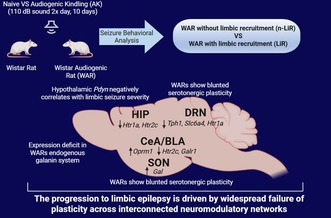

## Graphical abstract

To find molecular reasons why a genetic rat model (Wistar Audiogenic Rat, WAR) moves from sound‐triggered brainstem seizures to longer‐lasting limbic (temporal‐lobe) epilepsy after repeated loud‐noise exposure. WAR and normal Wistar rats received a 10‐day audiogenic kindling (twice‐daily loud noise). Brains were sampled to measure mRNA for serotonin‐related presynaptic components (dorsal raphe nucleus—DRN), serotonin receptors (basolateral and central amygdala—BLA and CeA—and hippocampus—HIP), galanin (GAL) and prodynorphin (PDYN) in the hypothalamus (SON) and their receptors in limbic areas. WARs were classified by behavior into limbic‐recruited (LiR) and non‐limbic (n‐LiR) groups. Normal rats showed strong upregulation of presynaptic serotonin genes in the dorsal raphe after repeated stimulation; WARs failed to mount this adaptive response. n‐LiR WARs increased amygdala serotonin receptors (a compensatory protective change); LiR WARs did not. WARs had increased hypothalamic galanin but reduced galanin receptor expression in limbic regions, limiting galanin's protective action. The opioid system was imbalanced in WARs: higher mu‐opioid receptor expression (pro‐convulsant) and lower prodynorphin (Pdyn); lower Pdyn correlated strongly with worse limbic seizures. Progression to limbic epilepsy in WARs is not due to a single defect but to a widespread failure of adaptive plasticity across multiple neuromodulatory systems (serotonin, galanin, dynorphin/opioids) and their limbic connections. Preventing or treating this epilepsy progression likely requires restoring network‐wide adaptive signaling rather than targeting one molecule.

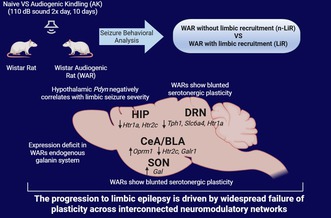

**Abbreviations**
5‐HT: Serotonin
AK: Audiogenic Kindling
BLA: Basolateral Amygdala
CeA: Central Amygdala
DRN: Dorsal Raphe Nucleus
Gal: Galanin
HIP: Hippocampus
LiR: Limbic‐Recruited Seizures
n‐LiR: Non‐Limbic‐Recruited Seizures
Pdyn: Prodynorphin
RRID: Research Resource Identifier
SON: Supraoptic Nucleus
WAR: Wistar Audiogenic Rat

## 1 Introduction

Epilepsy is a chronic neurological disorder defined by enduring susceptibility to generate epileptic seizures, affecting millions of individuals worldwide (World Health Organization 2023). This condition is characterized by transient symptoms arising from abnormal, excessive, or synchronous neuronal activity in the brain, with etiologies ranging from structural and metabolic to genetic factors (Berg et al. 2010). The consequences of chronic and uncontrolled seizures can lead to significant cognitive, locomotor, and psychosocial comorbidities that profoundly impact quality of life (Fisher et al. 2014). Thus, molecular studies that investigate the interaction between genetic background and the progressive development of seizures could aid in understanding the fundamental physiopathology of epileptogenesis and developing more effective diagnostics, prognostic, and therapeutic strategies for epileptic patients (Balestrini and Sisodiya 2018; Myers and Mefford 2015).

Genetically selected animal models provide an invaluable platform for dissecting these complex interactions. The Wistar audiogenic rats (WAR) strain is a well‐established model for reflex epilepsy, with selective inbreeding of Wistar rats for their susceptibility to seizures triggered by high‐intensity acoustic stimulation (Garcia‐Cairasco et al. 1996, 2017). The chronic acoustic stimulation, known as audiogenic kindling (AK) (Marescaux et al. 1987) can induce tonic–clonic seizures as an expression of the activation of mesencephalic brain structures, such as inferior colliculus, periaqueductal gray and the dorsal raphe nucleus (DRN) (Tupal and Faingold 2011). Additionally, it can lead to limbic seizures characterized by stereotyped automatisms, thus modeling the transition from brainstem‐driven events to temporal lobe‐like epilepsy, due to the recruitment of the limbic circuit, including structures such as the cortex, amygdala and hypothalamus (Dutra Moraes et al. 2000; Romcy‐Pereira and Garcia‐Cairasco 2003; Garcia‐Cairasco 2002; Valentim‐Lima et al. 2021).

The serotonergic (5‐HT) system, with its primary neuronal somas located in the DRN of the brainstem (Ptak et al. 2009), is a powerful modulator of neuronal excitability (Jacobs et al. 2002) and is widely proposed to have antiepileptic effects in the brain (Faingold 2004; Bagdy et al. 2007; Massey et al. 2021). Dysfunction within this system is implicated not only in seizure modulation but also in the pathophysiology of epilepsy‐related comorbidities and, critically, in Sudden Death in Epilepsy (SUDEP) (Devinsky et al. 2016; Patodia et al. 2022). Seizure activity can disrupt DRN neuron firing, and a compromised 5‐HT system is a leading hypothesis for the mechanisms underlying SUDEP (Cheng et al. 2022). In that scenario, it is interesting to state that WARs have reduced resting ventilation and ventilatory response to hypercapnia (7% CO_2_) and, that the number of chemically coded (Phox2b^+^/TH^−^, i.e., non‐catecholaminergic) retrotrapezoid nucleus (RTN) neurons, as well as the serotonergic innervation to the RTN (central chemoreceptor respiratory system), was reduced in WARs (Totola et al. 2017). Because WARs have additional cardiovascular alterations (Fazan Jr et al. 2011, 2015) and have an endogenous profile of stress (Umeoka et al. 2011), a natural consequence is that WARs were claimed as a unique rat model to study respiratory alterations associated to SUDEP (Hodges 2017).

Beyond the monoaminergic systems, neuropeptides act as potent endogenous modulators of neuronal networks, particularly under conditions of stress and network hyperexcitability. The chronic, repeated seizure induction characteristic of kindling protocols serves as a significant physiological stressor, engaging multiple neuromodulatory systems beyond those typically associated with epilepsy (Tupal and Faingold 2011). We recently demonstrated a significant imbalance in the hypothalamic vasopressin (*Avp*) and oxytocin (*Oxt*) RNA expression and hormone secretion in WAR (Valentim‐Lima et al. 2021). The magnocellular neurons (MCNs) responsible for producing and secreting these classic neurohypophysial neuropeptides also synthesize two other regulatory peptides, galanin (GAL) and dynorphin (DYN) (Villar et al. 1990). Galanin, acting through its receptors (*Galr1*, *Galr2*), is a well‐documented endogenous anticonvulsant, primarily by inhibiting presynaptic glutamate release in limbic structures like the hippocampus (Mazarati 2004) and amygdala (Gopalakrishnan et al. 2021; Mitsukawa et al. 2008). Clinical evidence has linked low brain GAL levels to seizure severity, suggesting its critical role in maintaining network stability (Somani et al. 2020). Similarly, dynorphin, an endogenous opioid peptide derived from the prodynorphin precursor (PDYN), also exerts powerful anticonvulsant effects, predominantly trough the activation of the κ opioid receptor (*Oprk1*) (Loacker et al. 2007; Schwarzer 2009). Gene therapy approaches to overexpress *Pdyn* have successfully suppressed seizures in animal models of temporal lobe epilepsy (Agostinho et al. 2019). Conversely, activation of μ opioid receptor (*Oprm1*) has been associated with pro‐convulsivant responses, highlighting a complex balance within the opioid system (Loacker et al. 2007; Romualdi et al. 1999).

We propose that the serotonergic, galaninergic, and opioidergic systems form a functionally integrated network that is critical for this adaptive response. These systems are linked anatomically and functionally: galanin is co‐expressed in and modulates the activity of 5‐HT neurons in the DRN (Mazarati et al. 2005); the 5‐HT system, in turn, is necessary for maintaining basal expression and regulation of the dynorphin system in the hippocampus (D'Addario et al. 2007); and galanin and dynorphin are co‐synthesized within the same magnocellular neurons of the hypothalamus (Villar et al. 1990), suggesting coordinated regulation. Furthermore, galanin is known to have an antagonistic interaction with the mu‐opioid system (Moreno et al. 2017). While these systems are individually recognized as important seizure modulators, their dynamic interplay and collective plastic changes in a genetic model of progressive seizure recruitment remain poorly understood.

The 5‐HT neurons from the DRN send direct projections to the limbic system, modulating the activity of hippocampus and amygdala neurons (Bombardi et al. 2021; Sengupta and Holmes 2019). MCNs of the SON send collateral projections to limbic areas, establishing a potential hypothalamo‐limbic axis for seizure modulation (Jiang‐Xie et al. 2019). The transition from brainstem to limbic seizures in the WAR model provides a unique opportunity to investigate the molecular adaptations or failures that govern this network‐level shift. Therefore, the present study was designed to test the hypothesis that the emergence of limbic seizures in WARs subjected to AK is driven by a network‐level failure of adaptive plasticity across these three critical interconnected neuromodulatory systems. We believe that an innate deficit in the capacity of these systems to respond to chronic seizure activity leads to a state of unchecked limbic hyperexcitability. To this end, we investigated AK‐induced changes in the mRNA expression of key genes related to 5‐HT, galanin, and dynorphin signaling in mesencephalic (DRN), hypothalamic (SON), and limbic (amygdala, hippocampus) structures.

## 2 Materials and Methods

### 2.1 Animals and Ethical Approval

Males 60–70 days old WAR (RRID: n/a; 250–280 g) and Wistar (RRID:RGD_13508588; 280–300 g) rats were obtained from the animal facility of the University of São Paulo, Ribeirão Preto campus. They were group‐housed in cages with four rats each. They had access to food and water *ad libitum*, were exposed to controlled light conditions (12/12 h) and maintained at room temperature (22°C ± 2°C) throughout the experiment. Animals were arbitrarily assigned to experimental groups. No specific randomization method was used. During the study, no animals were excluded or replaced. The study was conducted at the animal facility of the Physiology Department, Medical School of Ribeirão Preto, University of São Paulo (FMRP‐USP). Ethical approval for this study was obtained from the FMRP‐USP Animal Use Ethics Committee under protocol 242/2018, following the “Guide for the care and use of laboratory animals.”

### 2.2 Experimental Protocol: Audiogenic Kindling

The AK was performed as described by Dutra Moraes et al. (Dutra Moraes et al. 2000). WAR (*N* = 18) and Wistar (*N* = 12) rats were housed in acrylic cages with a chamber (32 × 30 × 30 cm) situated inside another similar structure, providing acoustic isolation (45 × 45 × 40 cm). The protocol consisted of three steps: (a) one minute to environmental adaptation; (b) one minute of exposure to a sound stimulus with a pre‐recorded school bell sound (120 dB) or until rats triggered mesencephalic convulsions; and (c) one minute inside of chamber before returning to the home cage. This protocol was performed twice a day (between 9:00–10:00 AM and 4:00–5:00 PM) for ten consecutive days, for a total of 20 stimulation sessions. All AK sessions were recorded for subsequent behavioral analysis. Quantification of the seizure severity index and limbic recruitment index was performed via offline analysis of video recordings. Each rat in the seizure scoring experiments was assigned a unique numerical code to mask treatment group identification, ensuring that analysts conducted unbiased evaluations of seizure severity and limbic recruitment indices without awareness of group assignments until after analysis was completed.

During the acoustic stimulation at step b, rats were evaluated and ranked for mesencephalic recruitment using the categorized seizure severity index (cSI) (Garcia‐Cairasco et al. 1996; Rossetti et al. 2006) and for limbic recruitment according to Racine's severity seizure index (Racine 1972). The cSI scale for seizures displayed by WAR exposed to acoustic stimulation ranged between 0 = no seizure; 1 = wild‐running; 2 = wild‐running + jumps; 3 = two wild‐running episodes followed; 4 = isolated tonic seizure; 5 = generalized tonic seizure; 6 = all of the above + opisthotonos; 7 = all of the above + hyperextension of the forelimbs; 8 = all of the above + hyperextension of the back limbs (Garcia‐Cairasco et al. 1996; Jiang‐Xie et al. 2019). The Racine score regarding the severity of displayed seizures by WAR ranges between 0 = immobility; 1 = facial automatism; 2 = cranial movement; 3 = forelimb tonus; 4 = rearing; 5 = rearing and falling (Rossetti et al. 2006).

**FIGURE 1:**
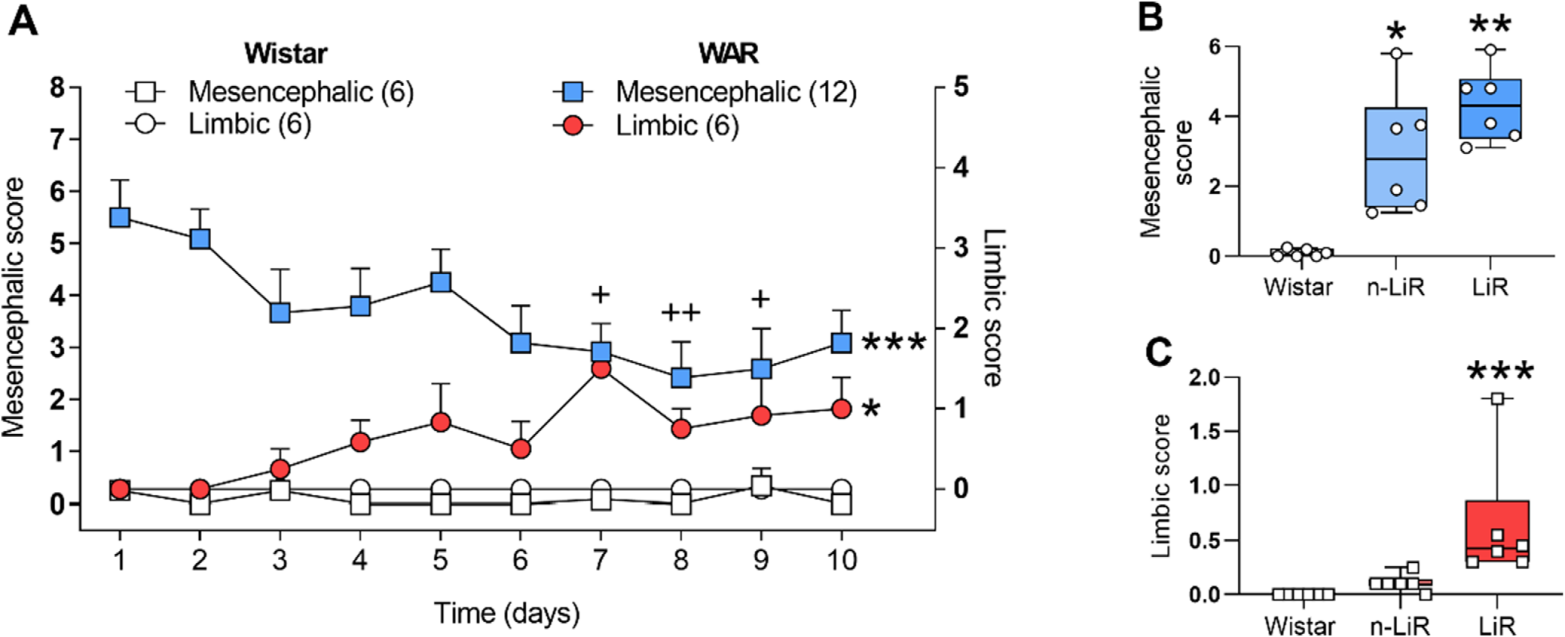
(A) Effects Caused by audiogenic kindling (AK) stimuli over days in Wistar and Wistar Audiogenic Rats (WARs) Based on mesencephalic and limbic seizure severity scores. Data were analyzed using the Friedman test with Dunn's post hoc test. *p < 0.05 indicates a significant difference between limbic and mesencephalic seizures. +*p* < 0.05 indicates a significant difference between AK during the stimulation days, compared to the first day. Error bars represent Mean ± SEM (B, C) Average mesencephalic and limbic scores in Wistar and WARs without (n‐LiR) and with limbic (LiR) seizures under AK. Data were analyzed using the Kruskal‐Wallis test followed by Dunn's post hoc test. *p < 0.05 indicates the difference between n‐LiR and LiR in WARs compared to the Wistars; **p < 0.01; ***p < 0.001; ++*p* < 0.01. Box plots display the median (center line), interquartile range (box), and minimum/maximum values (whiskers). All individual data points are shown.

Both Wistar and WAR groups were submitted to naive procedure (6 from each group) or AK (6 Wistar and 12 WAR). Control animals (termed ‘naïve’) were subjected to the same handling, transport, and placement in the acoustic chamber for identical durations as the AK groups, but the acoustic stimulus was not presented. The seizure profiles triggered by AK in WAR were next classified into limbic recruited (LiR) if at least three limbic seizures were observed after the sound stimulus or non‐limbic recruited (n‐LiR) if they showed fewer than three limbic seizures in all stimuli sections. The mesencephalic and limbic seizures are represented as the average scores (morning and afternoon) per day (Figure 1A) and the average scores along the 10 days (Figure 1B,C).

**FIGURE 2:**
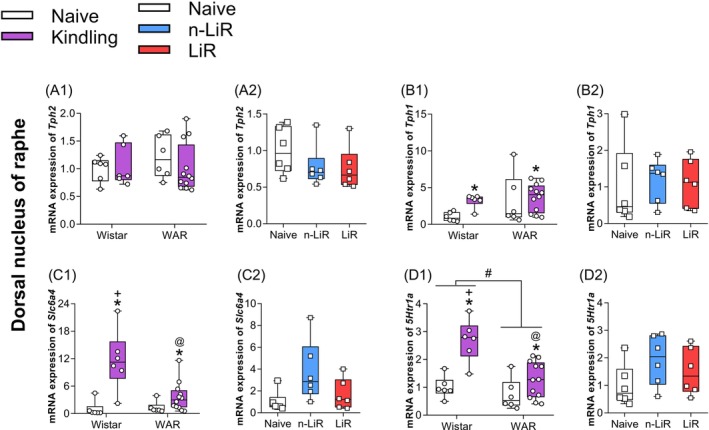
Effect of audiogenic kindling (AK) on messenger RNA (mRNA) expression in Dorsal Raphe Nucleus (DRN) of Tryptophan hydroxylase 2 (*Tph2*), Tryptophan hydroxylase 1 (*Tph1*), solute carrier family 6 (serotonin neurotransporter—*Slc6a4*) and serotonin receptor 1A (*Htr1a*) in Wistar and Wistar Audiogenic rats (WAR) (A1, B1, C1, D1). These data were analyzed by Two‐way ANOVA followed by Tukey's post‐test. We also analyzed whether AK stimuli trigger limbic recruitment (LiR) or not (n‐LiR) in WARs under AK versus naive rats on the mRNA expression of *Tph2, Tph1, Slc6a4* and *Htr1a* (A2, B2, C2, D2) in the DRN, analyzed by One‐way followed by the Tukey post‐test. #*p* < 0.05 WARs versus Wistars; *AK versus naive; +AK versus control; @WARs under AK versus Wistars. Box plots display the median (center line), interquartile range (box), and minimum/maximum values (whiskers). All individual data points are shown.

**FIGURE 3:**
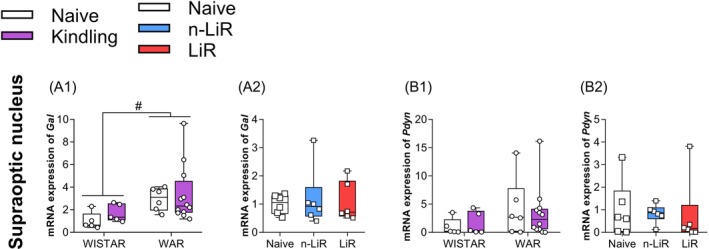
(A1, B1): Audiogenic kindling (AK) Effects on mRNA Expression in Supraoptic Nucleus of Galanin (*Gal*) and Prodynorphin (*Pdyn*). Analyzed by Two‐way ANOVA with Tukey's post‐test. # WARs versus Wistars. We also analyzed whether AK stimuli trigger limbic recruitment (LiR) or not (n‐LiR) in Wistar Audiogenic Rats (WARs) under AK versus naive rats on the mRNA expression of (A2, B2) *Gal* and *Pdyn* analyzed by One‐way followed by the Tukey post‐test. This data is expressed in Boxplot. #*p* < 0.05 WARs versus Wistars. Box plots display the median (center line), interquartile range (box), and minimum/maximum values (whiskers). All individual data points are shown.

### 2.3 Brain Tissue Collection and Micropunching

The rats were decapitated 16 h after the last control or AK protocol, without prior anesthesia, to prevent alterations in gene expression (Reed et al. 2009; Staib‐Lasarzik et al. 2014; Tanaka et al. 1989). The brains were collected, and rapidly frozen in dry ice before storage at −80°C. Following that, frozen brains were sliced (Leica Microsystems CM1850) in 60 μm thick sections for the SON and 100 μm for the DRN, central (CeA), and basolateral amygdala (BLA). The micropunch technique was then applied, using a 1 mm diameter needle for the specific collection of each brain nucleus. Subsequently, each brain section was stained with 0,1% toluidine blue and visualized under the microscope to confirm obtained region (Tupal and Faingold 2011). Any micropunch samples that did not correspond to the intended nuclei were excluded from gene expression evaluation. The collection of the hippocampus involved microdissection using a microtome blade. Subsequently, both micropunch and microdissected samples were stored in TRIzol reagent (Life Technologies, Waltham, MA, USA; cat. no. 15596026) at −80°C.

### 2.4 RNA Extraction, cDNA Synthesis, and qPCR

Total RNA was extracted by TRIzol protocol following the manufacturer's recommendations. RNA concentration and purity (260/280 ratio) were measured using a NanoDrop spectrophotometer (Thermo Scientific), with only samples exhibiting a 260/280 ratio between 1.8 and 2.1 proceeding to the next step. Subsequently, all samples were adjusted to an equal total RNA concentration and treated with a Dnase I Amp Grade kit (Life Technologies, Carlsbad, CA, USA) to eliminate genomic DNA. Following this step, cDNA was synthesized using a high‐capacity cDNA reverse transcription kit (Applied Biosystems, Beverly, MA, USA).

Gene expression was quantified using real‐time quantitative PCR (qPCR) on a QuantStudio 5 Real‐Time PCR System (Applied Biosystems). For TaqMan assays, pre‐designed probes were used for *Actb* (Rn00667869_m1), *Htr1a* (Rn00561409_s1), *Htr1b* (Rn01637747_S1), *Htr2a* (Rn00568473_m1) and *Htr2c* (Rn00562748_m1), *Slc6a4* (Rn00564737_m1), *Gal* (Rn00583681_m1), *Galr1* (Rn02132426_s1), *Galr2* (Rn00695901_g1), *Tph1* (Rn01476867_m1), *Tph2* (Rn00598017_m1), (Applied Biosystems, Beverly, MA, USA). For SYBR Green assays, custom primers were synthesized by Thermofisher for *Rpl19* (5′‐GCGTCTGCAGCCATGAGTA‐3′–5′‐TGGCATTGGCGATTTCGTTG‐3′), *Pdyn* (5′‐GGCTTCCTCTGTGGCACTTCT‐3′–5′‐TGTGGATGAGTGACCTGCGTG‐3′), *Oprm1* (5′‐ACACCGAAACTGGGAAGCCC‐3′—5′‐TCTTACATGGACCAGCCAGCA‐3′), *Oprk1* (5′‐GCACCAAAGTCAGGGAAGATGTG‐3′–5′‐AGCGCAGGATCATCAGGGTG‐3′). Primer efficiency was validated via standard curve with five points in a 1:2 factor of dilution, and confirmed to be between 90% and 110%. Relative gene expression was analyzed by the 2^−ΔΔCT^ method (Livak and Schmittgen 2001). The endogenous controls used were the *Actb* gene in the TaqMan assays and the *Rpl19* in the SYBR Green assays. Both genes used as endogenous controls demonstrate stable expression among the experimental conditions, with no significant differences observed between groups or conditions in the different brain nuclei studied.

### 2.5 Statistical Analysis

The data were submitted to the Shapiro–Wilk normality test. Non‐normally distributed data were either analyzed using the nonparametric Kruskal‐Wallis test or transformed into rankings before performing the two‐way ANOVA test (Hora and Conover 1984). The normal distribution data were analyzed using either one‐way or two‐way ANOVA. The progression of AK was analyzed using the Friedman test, a non‐parametric statistical test used to detect differences in treatments across multiple test attempts when the data do not meet the assumptions of normality. Two‐way ANOVA was used to assess the interaction between independent variables (strain and AK) and dependent variables. One‐way ANOVA or Kruskal‐Wallis tests were used, depending on the distribution of the data, to compare the difference between naive to n‐LiR or LiR. Subsequently to the ANOVA tests, Tukey's post‐test was applied, while the Kruskal‐Wallis and Friedman tests were followed by Dunn's post‐test for multiple comparisons. Spearman correlation analysis was applied to determine the monotonic relationship between the mesencephalic and limbic seizures, severity indexes, and gene expression in WARs after AK. The results are visually represented as a heatmap generated in RStudio to plot the matrix data, reporting the Spearman's ρ (rho) and the *p*‐values. A dendrogram was created based on the distance between the pairs using the Euclidean method and clustered by average. No statistical test for outliers was performed, and no data points were excluded from the analyses. No a priori sample size calculation was performed. The number of animals per group (*n* = 6 for molecular analyses) was determined based on previous studies from our group and others using this model (Valentim‐Lima et al. 2021), which have consistently shown sufficient statistical power to detect meaningful biological effects.

All other statistical analyses and graphical representations were performed by using GraphPad Prism 9.0 (Los Angeles, USA). The results are presented as medians, upper, and lower quartiles, minimum and maximum values in a boxplot format, considering results significant at *p* < 0.05.

## 3 Results

### 3.1 Audiogenic Kindling Drives a Shift From Mesencephalic to Limbic Seizure Dominance in WARs

The ten‐day audiogenic kindling (AK) protocol induced distinct behavioral responses in Wistar and WAR strains. As expected, control Wistar rats did not exhibit any significant seizure activity, showing negligible scores on both mesencephalic and limbic scales throughout the protocol. In contrast, all WARs displayed robust seizure responses to the acoustic stimuli.

The temporal dynamics of seizure expression revealed a clear pattern of network reorganization over the 10 days of kindling (Figure 1A). On the first day of stimulation, WARs exhibited high mesencephalic seizure scores, indicative of brainstem circuit recruitment. These scores progressively decreased over the course of the protocol, with a statistically significant reduction (*χ* ^2^(9) = 29.61, *p* = 0.0005) observed on Days 7 (*p* = 0.00430), 8 (*p* = 0.0072), and 9 (*p* = 0.0159) compared to Day 1. Concurrently, a subset of WARs began to display limbic seizures around Day 3 of the protocol and their severity scores progressively increased, reaching a peak around Day 7 (Figure 1A).

Based on this phenotype, the WAR cohort was divided into two subgroups: 50% (*n* = 6) were classified as limbic recruited (LiR), showing a significant increase in limbic scores over the 10 days (*χ* ^2^(9) = 17.96, *p* = 0.0356), while the remaining 50% (*n* = 6) were classified as non‐limbic recruited (n‐LiR) exhibiting minimal to no limbic seizure activity.

Analysis of the average seizure scores over the entire 10‐day protocol confirmed these observations. Both LiR and n‐LiR WARs had significantly higher average mesencephalic scores than kindled Wistar rats (H(2) = 12.39, *p* = 0.0001; Dunn's post‐test: *p* = 0.0019 and *p* = 0.0441, respectively), with no significant differences between the two WAR subgroups (Figure 1B). As expected by their classification, the average limbic score over the 10 days of AK demonstrated differences among the groups (H(2) = 14.94, *p* < 0.0001), with post‐tests indicating a significant increase in WARs with LIR compared to Wistars (*p* = 0.0003) (Figure 1C).

### 3.2 WARs Exhibit Blunted Pre‐Synaptic Serotonergic Plasticity in the Dorsal Raphe Nucleus Following AK

To investigate the molecular basis of this seizure progression, we first examined the expression of key genes involved in serotonin (5‐HT) synthesis and regulation within the Dorsal Raphe Nucleus (DRN), the primary source of forebrain 5‐HT. Expression of Tryptophan hydroxylase 2 (*Tph2*), the canonical rate‐limiting enzyme for neuronal 5‐HT synthesis, showed no significant differences related to strain, AK, or limbic recruitment status (Figure 2A1,A2). This suggests that the basal capacity for 5‐HT production is not inherently different in WARs.

In contrast, the expression of genes associated with the plastic and stress‐responsive regulation of the 5‐HT system revealed a profound deficit in WARs. Tryptophan hydroxylase 1 (*Tph1*) an isoform implicated in stress‐induced 5‐HT synthesis, was significantly upregulated by AK overall (*F*_(1.26)_ = 7.274, *p* = 0.0121), (Figure 2B1). This effect was driven almost entirely by the control Wistar rats, which showed a nearly 3‐fold increase in *Tph1* mRNA after kindling. WARs, however, exhibited a completely blunted response, with no significant change in *Tph1* expression following AK (Figure 2B2).

A similar pattern of failed adaptation was observed for the serotonin transporter (*Slc6a4*, SERT) and the 5‐HT1A autoreceptor (*Htr1a*), both of which are critical for regulating synaptic 5‐HT levels and neuronal firing. For *Slc6a4*, two‐way ANOVA revealed a significant effect of AK (*F*_(1.26)_ = 27.21, *p* < 0.0001) and a significant interaction between AK and strain (*F*_(1.26)_ = 8.041, *p* = 0.0087). For *Htr1a*, analysis showed significant main effects for both strain (*F*_(1.26)_ = 9.135, *p* = 0.0056) and AK (*F*_(1.26)_ = 15.68, *p* = 0.0005). In Wistar rats, post hoc analysis showed AK induced a substantial increase in the expression of both *Slc6a4* (*p* < 0.0001) and *Htr1a* (*p* = 0.0085), indicating a robust homeostatic response to the chronic stimulation. In WARs, this adaptive response was severely attenuated for both genes (*Slc6a4*: *p* = 0.0461; *Htr1a*: *p* = 0.013) (Figure 2C1,D1). When comparing the WAR subgroups, there was a trend for increased *Slc6a4* in n‐LiR compared to naïve controls (*p* = 0.058), but no significant changes were noted between n‐LiR, LiR, and naive WARs for *Tph1* or *Htr1a* (Figure 2C2,D2). This collective failure to upregulate key components of the 5‐HT synthesis‐reuptake‐feedback loop in the DRN points to an innate deficit in the adaptive capacity of the serotonergic system in the WAR strain, when faced with a chronic epileptogenic challenge.

### 3.3 The Hypothalamic Galaninergic, but Not Opioidergic, System Is Basally Altered in WARs

As we have recently demonstrated significant changes in *Avp* and *Oxt* expression in the SON and PVN of WARs submitted to AK (Valentim‐Lima et al. 2021), this study also evaluates the expression of the mRNAs coding *Gal* and *Pdyn* in SON, given their high production by the MCNs (Villar et al. 1990) and their relevance to epilepsy physiopathology (Devinsky et al. 2016; Somani et al. 2020; Agostinho et al. 2019; Mazarati et al. 2001). We observed a significant increase in SON *Gal* expression in WARs compared to Wistar (*F*_(1.24)_ = 12.92, *p* = 0.0015), with WARs exhibiting significantly higher basal levels of *Gal* mRNA compared to Wistar rats, irrespective of the kindling procedure (Figure 3A1). Additionally, n‐LiR and LiR WARs after AK exhibited similar levels of SON *Gal* expression when compared to naive (Figure 3A2). This inherent overexpression of an anticonvulsant peptide in a seizure‐prone strain suggests the presence of a constitutive, yet potentially insufficient, compensatory mechanism.

In contrast, the expression of *Pdyn* mRNA in the SON showed no differences based on strain, AK, or limbic recruitment status (Figure 3B1,B2). This finding isolates the basal alteration specifically to the galaninergic system within the hypothalamus.

### 3.4 Post‐Synaptic Serotonin Receptor Expression Is Differentially Regulated in the Amygdala of Kindled WARs

**FIGURE 4:**
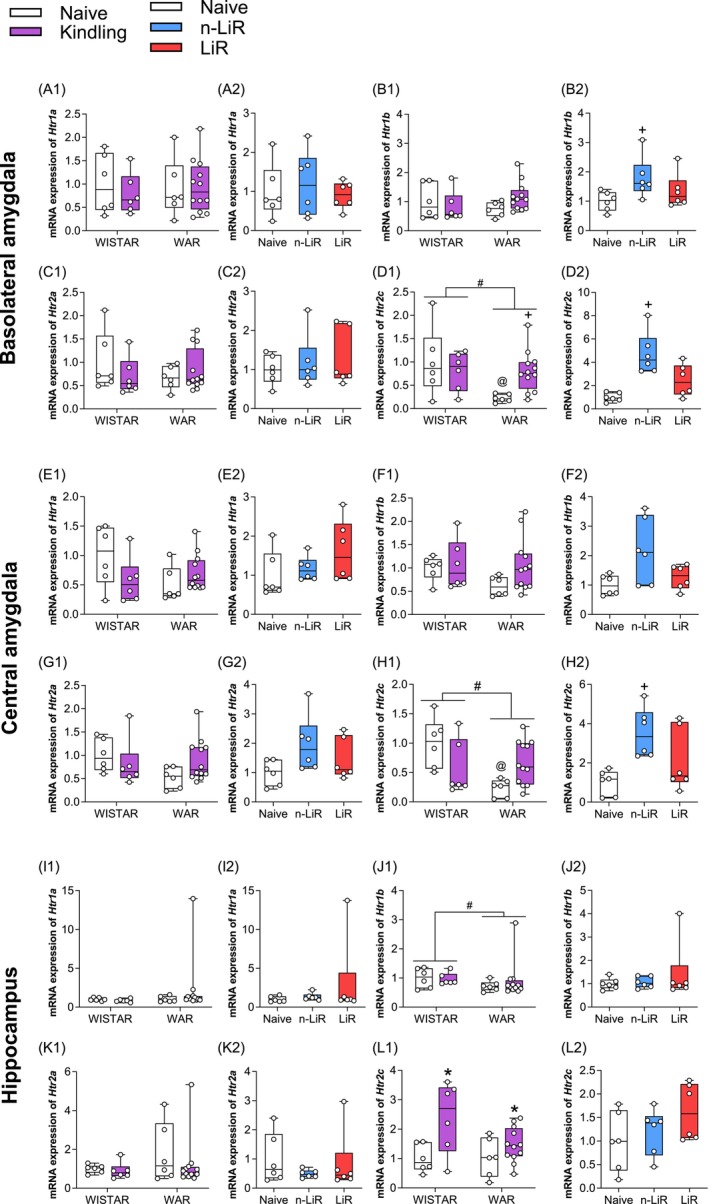
Effect of audiogenic kindling (AK) on messenger RNA (mRNA) expression in the basolateral (A1—D2) and central amygdala (E1—H2) and hippocampus (I1—L2) of serotonin post synaptic receptors 1A (*Htr1a*), 1B (*Htr1b*), 2A (*Htr2a*), and 2C (*Htr2c*) in Wistar and Wistar Audiogenic rats (WAR). These data were analyzed by Two‐way ANOVA followed by Tukey's post‐test or One‐way followed by the Tukey post‐test. #*p* < 0.05 WARs versus Wistars; *AK versus naive; +AK versus control; @WARs under AK versus Wistars. Box plots display the median (center line), interquartile range (box), and minimum/maximum values (whiskers). All individual data points are shown.

Given the impaired pre‐synaptic 5‐HT plasticity in the DRN of WARs, we hypothesized that differential post‐synaptic receptor regulation in limbic target areas might determine the progression to limbic seizures. We measured the mRNA expression of four 5‐HT receptor subtypes in the basolateral amygdala (BLA), central amygdala (CeA), and hippocampus (HIP) (Figure 4).

In the BLA, the most significant finding was a differential regulation of *Htr1b* and *Htr2c* that correlated with the resistance to limbic recruitment after AK. Specifically, n‐LiR WARs showed a significant increase in *Htr1b* mRNA compared to their naive counterparts (*p* = 0.0331) (Figure 4B2). A similar, even more pronounced pattern was observed for *Htr2c*. In the BLA, WARs exhibited an attenuated expression of *Htr2c* mRNA compared to Wistars (*F*_(1.26)_ = 5.195, *p* = 0.0311). In the CeA, analysis also showed a main effect of strain (*F*_(1.26)_ = 4.806, *p* = 0.0375) and a significant interaction (*F*_(1.26)_ = 10.07, *p* = 0.0039). Post hoc analysis revealed that basal expression of *Htr2c* was significantly lower in both the BLA (*p* = 0.0450) and CeA (*p* = 0.0077) of naive WARs compared to naive Wistars, indicating a potential innate vulnerability. Following AK, n‐LiR rats showed a robust compensatory response, significantly upregulating *Htr2c* expression in both the BLA (*p* = 0.0029) and CeA (*p* = 0.0077) compared to naive WARs (Figure 4D2,H2). Critically, this adaptive upregulation of both Htr1b and Htr2c was absent in the LiR rats, which progressed to severe limbic seizures. The large dispersion observed in the LiR group for some measures likely reflect the inherent biological heterogeneity in the severity of the kindled phenotype among individual animals, a known characteristic of this genetic model.

These data suggest that the capacity to upregulate post‐synaptic 5‐HT receptor expression in the amygdala may serve as a molecular correlate of resilience against limbic network recruitment. Other 5‐HT receptors showed less consistent changes. For instance, hippocampal *Htr1b* was lower in WARs overall (*F*_(1.26)_ = 6.364, *p* = 0.0181), and hippocampal Htr2c was increased by AK in both strains (*F*_(1.26)_ = 10.43, *p* = 0.003), but these changes did not distinguish between the n‐LiR and LiR phenotypes (Figure 4J1,L1).

### 3.5 AK Induces a Pro‐Convulsant Shift in Limbic Opioid and Galanin Receptor Profiles

**FIGURE 5:**
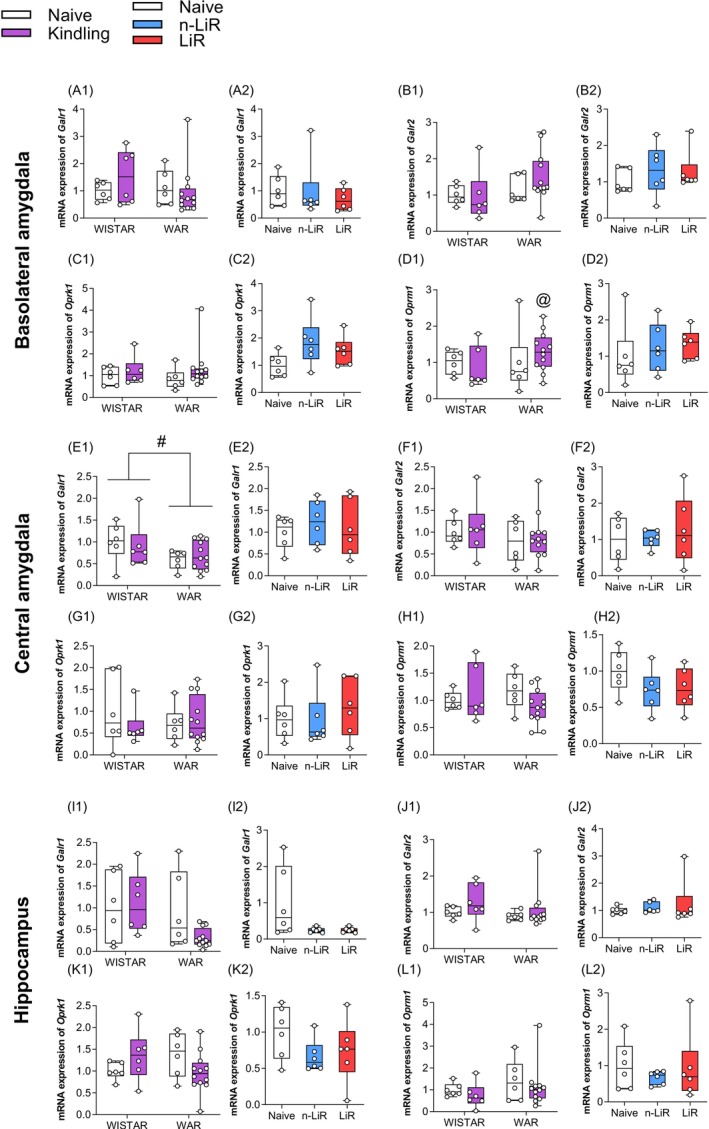
Effect of audiogenic kindling (AK) on messenger RNA (mRNA) expression in the basolateral amygdala (A1—D2), central amygdala (E1—H2), and hippocampus (I1—L2) of galanin (*Galr1* and *Galr2*) and dynorphin (*Oprm1* and *Oprk1*) receptors in Wistar and Wistar Audiogenic Rats (WAR). These data were analyzed by two‐way ANOVA followed by Tukey's post‐test or one‐way followed by the Tukey post‐test. #*p* < 0.05 WARs versus Wistars. Box plots display the median (center line), interquartile range (box), and minimum/maximum values (whiskers). All individual data points are shown.

To complement the analysis of hypothalamic neuropeptide expression, we measured their corresponding receptors in the same limbic structures (Figure 5). This revealed a maladaptive shift in receptor profiles, particularly in WARs.

The paradoxical overexpression of hypothalamic *Gal* in WARs was met with a downstream receptor deficit. In the CeA, WARs exhibited significantly lower basal expression of the galanin receptor 1 (*Galr1*) compared to Wistars (*F*_(1.26)_ = 4662, *p* = 0.0403) (Figure 5E1). This finding suggests that the compensatory increase in galanin ligand production may be rendered ineffective by a reduced number of its primary anticonvulsant receptor in a key limbic structure. No significant changes were observed for *Galr2* in any region.

The opioid system showed a different form of maladaptive plasticity. There were no significant changes in the expression of the primarily anticonvulsant kappa‐opioid receptor (*Oprk1*) in any group or region. However, in the BLA, there was a significant interaction effect for the mu‐opioid receptor (*Oprm1*) (*F*_(1.26)_ = 6.213, *p* = 0.0194), which has been linked to pro‐convulsant effects. Post hoc analysis revealed that this was driven by a significant increase in *Oprm1* expression in WARs subjected to AK (*p* = 0.0308) (Figure 5C1). This AK‐induced upregulation of a potentially pro‐convulsant receptor, in the absence of any change in its anticonvulsant counterpart, suggests that chronic seizure activity in the genetically susceptible strain drives a shift in the opioid system's balance towards a state that may facilitate, rather than suppress, future seizures.

### 3.6 Correlation Network Analysis Reveals a Negative Relationship Between Hypothalamic Prodynorphin and Limbic Seizure Recruitment

**FIGURE 6:**
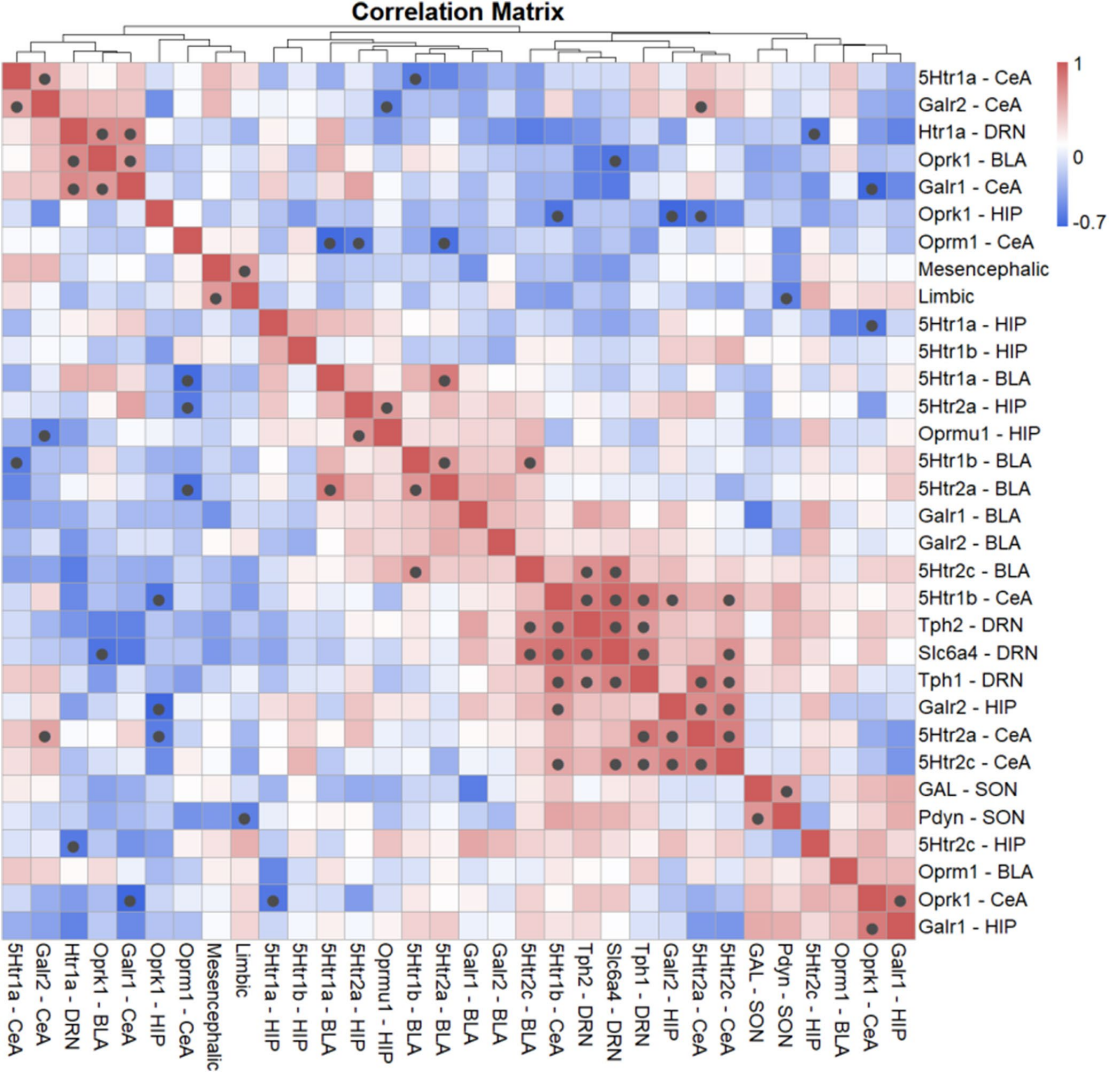
Correlation heatmap between all genes, structures and parameters evaluated, reporting Spearman correlation coefficients and *p* values (set as *p <* 0.05) for each comparison represented by black circle. The bar on the right side of the map indicates the color legend of the *rho* pairwise correlation. A *p* < 0.05 was accepted as significant. *p* > 0.10 are presented in white squares.

To synthesize these molecular findings and link them directly to the behavioral phenotypes, we performed a Spearman correlation analysis between all measured gene expression levels, and the seizure severity scores in the kindled WARs (*n* = 12). The results are visualized in a heatmap (Figure 6), which identified 39 significant monotonic relationships (*p* ≤ 0.05), providing a snapshot of the coordinated (or uncoordinated) molecular response to kindling.

Several key relationships emerged from the data. First, corroborating the behavioral observations, the average mesencephalic and limbic seizure scores were positively correlated (*r* = 0.652, *p* = 0.008), confirming that animals with stronger overall mean values of brainstem seizures scales, were more likely to develop limbic seizures. Second, within the DRN, the expression of the serotonergic genes *Tph1* and *Slc6a4* were strongly and positively correlated (*r* = 0.804, *p* = 0.002), suggesting a coordinated, albeit blunted, transcriptional program. Third, a significant positive correlation was found between the hypothalamic neuropeptides *Gal* and *Pdyn* (*r* = 0.678, *p* = 0.015), hinting at a potential co‐regulation of these two anticonvulsant systems at their source.

The most critical finding linking gene expression to phenotype was the relationship between the hypothalamic opioid system and limbic seizure severity. Despite no significant difference in the mean expression of *Pdyn* between groups, the correlation analysis revealed a highly significant, strong negative relationship between *Pdyn* mRNA levels in the SON and the average limbic seizure score (*r* = −0.619, *p* = 0.032). This indicates that individual WARs that were able to maintain higher levels of *Pdyn* expression in the hypothalamus experienced significantly less severe limbic seizures. This finding provides the strongest evidence in this study for a specific endogenous anticonvulsant mechanism.

Finally, highlighting inter‐system dynamics, a strong negative correlation was found between the inhibitory serotonin receptor *Htr1a* in the BLA and the potentially pro‐convulsant opioid receptor *Oprm1* in the CeA (*r* = −0.734, *p* = 0.007), suggesting a functional antagonism between protective and maladaptive signaling pathways.

## 4 Discussion

The present study sought to unravel the molecular mechanisms underlying the progression from brainstem‐dependent to limbic‐recruited seizures in the WAR strain, a genetic model of epilepsy (Garcia‐Cairasco et al. 2017). By employing an AK protocol (20 stimuli, 2 per day) and performing a detailed analysis of gene expression in key neuromodulatory circuits, our findings reveal a complex, network‐level failure of adaptive plasticity. The data point not to a single deficient gene, but to a tripartite dysregulation across the serotonergic, galaninergic, and opioidergic systems that collectively lowers the threshold for limbic network recruitment in genetically susceptible WARs.

Both seizure profiles (mesencephalic and limbic) are associated with an increased risk of death, primarily associated with breathing complications, such as sudden unexpected death (SUDEP) (Devinsky et al. 2016; Totola et al. 2017; Murugesan et al. 2018; Thurman et al. 2014, 2017). Respiratory impairments, including apnea, represent potential more severe acute consequences immediately following seizures in WARs (Fazan Jr et al. 2011; Garcia‐Cairasco 2002). Acute audiogenic stimulation typically induces mesencephalic seizures in WARs, allowing observation of respiratory consequences. In contrast, the chronic audiogenic stimulation (AK) results in LiR, inducing behavioral manifestations related to the called limbic seizures (Garcia‐Cairasco et al. 1996). In the current study, approximately half of the WARs submitted to AK exhibit limbic seizures, where limbic structures overlap with mesencephalic ones (Garcia‐Cairasco et al. 1996; Valentim‐Lima et al. 2021; Garcia‐Cairasco 2002). Our data align with previous evidence, revealing a high mesencephalic recruitment score on the first day of AK in WARs. However, the limbic recruitment score only emerges after the third day of AK. Throughout the 10 days of AK, there is a progressive reduction in the mesencephalic score in all WARs, while the limbic score progressively increases from Days 3 to 10 in 50% of the WARs, classified as limbic recruited, usually in a mirror image, when the behavioral scales are seen. Mesencephalic structures demonstrate to be those needed in the origin of acute audiogenic seizures, and additionally central to controlling the activation of limbic structures and subsequent behavior manifestations associated with limbic seizures both in WARs (Garcia‐Cairasco et al. 1996; Dutra Moraes et al. 2000) and in Genetically Epilepsy‐Prone rats, an analogous audiogenic susceptible strain derived from Sprague–Dawley progenitors (Merrill et al. 2005). The distinct patterns of mesencephalic and limbic recruitment, coupled with significant positive correlation between mesencephalic and limbic scores, underscore the interconnected nature of these responses.

Previous studies have shown altered 5HT neurotransmission at the retrotrapezoid nucleus in the central respiratory structures in WARs (Totola et al. 2017). In consonance with that, in the current study, the mRNA expression results converge to a coherent picture of systemic failure. First, the serotonergic system in WARs demonstrates lack of pre‐synaptic plasticity in the Dorsal Raphe Nucleus (DRN). Faced with the chronic stress of AK, WARs fail to mount the adaptive upregulation of key regulatory genes (*Tph1*, *Slc6a4*, *Htr1a*) observed in control Wistar rats. This pre‐synaptic deficit is compounded by a failure of post‐synaptic compensation in the amygdala of the most vulnerable animals (the LiR group). Second, the galaninergic system exhibits a basal signaling deficit. WARs show a paradoxical, likely compensatory, overexpression of hypothalamic *Gal* mRNA, but this is met with a constitutive reduction of its primary anticonvulsant receptor, *Galr1*, in the central amygdala, likely rendering the system ineffective. Third, the opioid system undergoes a maladaptive shift. The protective dynorphin system, as revealed by correlation analysis, appears to weaken in animals that develop severe limbic seizures, while the potentially pro‐convulsant mu‐opioid receptor, *Oprm1*, is pathologically upregulated by seizure activity in the basolateral amygdala. Together, these findings suggest a potential positive feedback loop of maladaptive plasticity where an innate inability to adapt to seizure activity drives further maladaptive changes, creating a state of progressive limbic hyperexcitability.

The serotonergic system is a critical regulator of whole‐brain excitability and homeostasis (Merrill et al. 2005; Patodia et al. 2018). Our finding that WARs exhibit a blunted transcriptional response in the DRN following AK is central to understanding their phenotype. While *Tph2* is the primary enzyme for constitutive 5‐HT synthesis in the brain (Austin and O'Donnell 2008; Ehret et al. 1987), *Tph1* expression in the DRN has been shown to be highly responsive to chronic stress (Chen et al. 2017; Abumaria et al. 2008). The robust upregulation of *Tph1*, along with the transporter (*Slc6a4*) and the autoreceptor (*Htr1a*), in Wistar rats represents a normal, adaptive homeostatic response to the chronic stressor of AK. The failure of WARs to mount this response points to a fundamental deficit in their central stress‐response machinery. This is consistent with previous reports of morphofunctional dysregulations in the hypothalamic–pituitary–adrenal (HPA) axis of the WAR strain (Umeoka et al. 2011; Abumaria et al. 2008; Godoy and Garcia‐Cairasco 2021). This suggests that the genetic susceptibility to seizures in WARs may be mechanistically intertwined with an impaired ability to cope with chronic stress at the molecular level within the brain's primary 5‐HT nucleus. This impaired plasticity would logically compromise the ability of the 5‐HT system to exert its well‐known anticonvulsant effects (Bagdy et al. 2007).

The relationship between pre‐synaptic components of the 5‐HTergic system, which are impaired in acoustic seizure animal strains such as WARs, and seizures associated with compromised respiratory behavior is demonstrated by several studies (Totola et al. 2017; Granjeiro et al. 2016). In our analysis, the downstream consequences of this pre‐synaptic failure are evident in the amygdala, a key gatekeeper for limbic seizure generalization. The observation that n‐LiR rats selectively upregulate the post‐synaptic receptors *Htr1b* and *Htr2c* in the amygdala is particularly insightful. This suggests a successful post‐synaptic compensatory mechanism by increasing the sensitivity of amygdala neurons to the limited 5‐HT that is available (Cheng et al. 2022), these animals may effectively bolster the inhibitory tone in this critical structure, preventing seizure spread. The LiR rats, which lack this post‐synaptic plasticity, are left vulnerable. The reduced basal expression and failed upregulation of Htr2c in LiR rats is especially noteworthy. Genetic deletion or dysfunction of the Htr2c receptor is linked to increased susceptibility to audiogenic seizures and is a feature of some SUDEP models (Brennan et al. 1997; Massey et al. 2021; Tecott et al. 1995). The overall dysfunction of the 5‐HT system in WARs, from impaired pre‐synaptic plasticity in the DRN to failed post‐synaptic adaptation in the amygdala, not only provides a mechanism for their seizure phenotype but also strongly implies an elevated risk for SUDEP, as the DRN is indispensable for respiratory and autonomic control during and after seizures (Devinsky et al. 2016; Totola et al. 2017; Thurman et al. 2017).

The WAR's hypothalamic neurohypophyseal system is also imbalanced, as previously demonstrated (Valentim‐Lima et al. 2021). This area is a crucial source of GAL and DYN produced by MNCs (Jacoby et al. 2002), besides its projections to important limbic regions such as the hippocampus and amygdala (Hodges 2017; Sengupta and Holmes 2019). The finding that WARs have constitutively higher levels of *Gal* mRNA in the SON is, at first glance, a paradox, since this peptide is considered a potent anticonvulsant (Villar et al. 1990; Hora and Conover 1984). This might be most likely a compensatory attempt to counteract the brain's innate hyperexcitability. This unsuccessful response is revealed by the corresponding decrease in the expression of its receptor, *Galr1*, in the CA nucleus. This “signaling deficit” is an illustration of how measuring only a ligand or a receptor in isolation can be misleading. The brain's attempt to increase the protective galanin signal is effectively short‐circuited by a lack of downstream targets. The anticonvulsant effects of galanin are known to be mediated primarily through *Galr1* activation, and *Galr1* knockout mice consequently show increased seizure susceptibility (Landry et al. 2003; Fetissov et al. 2003). Thus, the combination of high ligand and low receptor expression in WARs represents a clear molecular pathology.

The opioid system, in contrast, appears to be driven into a maladaptive state by the seizure activity itself. The most robust evidence for a protective role of dynorphin comes from our correlation analysis, which showed that because WARs are able to maintain higher hypothalamic *Pdyn* expression, they had significantly less severe limbic seizures. This aligns perfectly with extensive literature demonstrating the powerful anticonvulsant effects of the dynorphin/Oprk1 system (Somani et al. 2020; Schwarzer 2009). The fact that overall Oprk1 expression did not change suggests that the limiting factor may be ligand availability. In contrast, the expression of the mu‐opioid receptor, *Oprm1*, was significantly increased by AK in the BLA of WARs. Although Oprm1's role in epilepsy is not yet fully understood, it has been implicated in epilepsy disorders (Somani et al. 2020; Wang et al. 2017), and further investigation is needed to clarify its function and potential role in mediating the protective effects of DYN in seizure modulation. Therefore, chronic seizures in this genetic model appear to drive a pathological form of plasticity, actively upregulating a receptor system that may facilitate further seizures, creating a dangerous positive feedback loop.

The correlation analysis (Figure 6) provides a frame into the malfunction of coordinated regulation across these systems. It is crucial to distinguish the temporal dynamics of kindling (Figure 1), where mesencephalic scores wane as limbic seizures emerge, from the overall seizure propensity of individual animals. The significant positive correlation found is between the average mesencephalic and limbic scores for each rat over the entire protocol. This clarifies that an animal's underlying susceptibility to severe brainstem seizures is a strong predictor for the subsequent development of a more severe limbic epilepsy phenotype, rather than implying both seizure scales increase simultaneously. Within individual systems, we see evidence of coordinated transcriptional programs, such as the tight positive correlation between Tph1 and Slc6a4 in the DRN. However, they are the relationships between systems that are most revealing. The negative correlation between the protective hypothalamic peptide Pdyn and limbic seizure severity identifies it as a key suppressor of epileptogenesis. Furthermore, the negative relationship between the inhibitory *Htr1a* receptor and the pro‐convulsant *Oprm1* receptor in the amygdala suggests a critical tipping point in the balance of excitation and inhibition. Animals that fail to maintain protective serotonergic and dynorphinergic tone appear to upregulate maladaptive opioidergic signaling, creating a feed‐forward loop of hyperexcitability. These findings highlight that the transition to limbic epilepsy in WARs is not due to a single failure but to a collapse of the integrated network that normally maintains brain homeostasis.

Although this study provides a comprehensive snapshot of transcriptional changes, it has inherent limitations. A primary limitation is its reliance on mRNA expression data. While transcriptional changes provide valuable insight into cellular responses, they do not always directly correlate with protein levels or functional activity. For instance, inferring pre‐ or post‐synaptic changes based solely on gene expression in a whole nucleus punch is speculative, as receptors like 5‐HT1A are expressed on multiple cell types and in different subcellular compartments. Future studies employing techniques such as Western blotting, immunohistochemistry, or receptor binding assays are necessary to confirm these findings at the protein level. Furthermore, the characterization of seizure progression was based on behavioral scoring. While this method is well‐established for the WAR model, the absence of electrographic recordings (EEG) precludes a definitive analysis of seizure onset, propagation pathways, and subclinical activity. Our interpretations of ‘limbic recruitment’ should therefore be considered within the context of this behavioral framework. Finally, our study is observational and identifies strong correlations between gene expression patterns and seizure phenotype. However, these associations do not establish causality. Future work involving pharmacological or genetic manipulation of these specific targets, paired with functional readouts, would be required to determine their causal role in the epileptogenic process.

In conclusion, this study provides a multi‐faceted view of the molecular correlates for the progression of seizures in the WAR genetic epilepsy model. Our findings demonstrate that the transition from brainstem to limbic epilepsy, usually seen as video/behavior/EEG alterations in critical nodes of mesencephalic‐limbic networks (Dutra Moraes et al. 2000), for a comprehensive review, see (Garcia‐Cairasco et al. 2017), is not the result of a single gene defect but rather a systemic failure of adaptive plasticity across a network of crucial neuromodulatory systems. We identified a core deficit in the adaptive capacity of the pre‐synaptic serotonergic system, a paradoxical and ineffective compensatory response in the galaninergic system, and a maladaptive, pro‐convulsant shift in the opioid system. The strong negative correlation between hypothalamic dynorphin expression and limbic seizure severity highlights this neuropeptide as a critical endogenous brake on limbic hyperexcitability. These results underscore the importance of viewing epilepsy through a network lens and reveal novel, interconnected molecular targets, indeed signatures, including the *Htr2c*, *Galr1*, and *Oprm1* receptors for the development of future therapeutic strategies aimed at restoring homeostatic plasticity in the epileptic brain.

## Author Contributions

**Tays Araújo Camilo:** writing – original draft, writing – review and editing, formal analysis, data curation, validation, methodology. **Evandro Valentim‐Lima:** writing – original draft, writing – review and editing, methodology, validation, formal analysis, data curation. **José Antônio Cortes de Oliveira:** writing – review and editing, investigation. **Norberto Garcia‐Cairasco:** conceptualization, investigation, writing – review and editing, formal analysis, supervision. **Luís Carlos Reis:** writing – review and editing, investigation. **João Victor Nani:** writing – review and editing, investigation, methodology, formal analysis, data curation. **André de Souza Mecawi:** supervision, resources, formal analysis, project administration, writing – review and editing, writing – original draft, funding acquisition, visualization, validation, conceptualization, investigation.

## Funding

Coordenação de Aperfeiçoamento de Pessoal de Nível Superior—Brazil (CAPES, Finance Code 001). This work was funded by Fundação de Amparo à Pesquisa do Estado de São Paulo (FAPESP, grants 2014/50891‐1 and 2019/05957‐8 to NGC; 2024/15733‐8 to JVN and 2019/27581‐0, 2024/01620‐7 to ASM) and Conselho Nacional de Desenvolvimento Científico e Tecnológico (CNPq, grants 465 458/2014‐9 and 408 707/2018‐6 to NGC; and 315 564/2023‐7, 421 434/2023‐6 and 309 882/2020‐6).

## Conflicts of Interest

The authors declare no conflicts of interest.

## Data Availability

The data that support the findings of this study are available from the corresponding author upon reasonable request.
